# Retinoic acid promotes expression of germline-specific genes in chicken blastoderm cells by stimulating Smad1/5 phosphorylation in a feeder-free culture system

**DOI:** 10.1186/s12896-017-0332-y

**Published:** 2017-02-20

**Authors:** Xiaochuan Tang, Shiyong Xu, Hongpeng Zhang, Qing Chen, Rongyang Li, Wangjun Wu, Minli Yu, Honglin Liu

**Affiliations:** 1 College of Animal Science and Technology, Nanjing Agricultural University, Nanjing, 210095 People’s Republic of China; 2 College of Animal Science and Technology, Jingling Institute of Technology, Nanjing, 210095 People’s Republic of China

**Keywords:** Chicken blastodermal cells, E8 medium, Retinoic acid, Germ cell differentiation, Smad1/5 phosphorylation, Growth factor

## Abstract

**Background:**

Producing transgenic chickens with chicken blastodermal cells (cBCs) is inefficient due to the extremely low germline transmission capacity of cBCs. As chicken primordial germ cells (PGCs) have been reported as an efficient method for producing transgenic chickens, the inefficiency of cBCs could potentially be resolved by inducing them to differentiate into germ cells. However, whether chemical inducers are able to enhance cBCs germline competence in vitro is unknown and the molecular mechanisms of differentiation of chicken pluripotent cells into germ cells are poorly understood.

**Results:**

We cultured cBCs with a monolayer morphology in E8 medium, a xeno- and feeder-free medium. We showed that retinoic acid (RA) treatment increased expression of germ cell-specific genes in cBCs. Using western blot, we determined that RA stimulated Smad1/5 phosphorylation. Moreover, Smad1/5 activation regulates the expression of germ cell-specific genes, as co-treatment with a Smad1/5 phosphorylation inhibitor or activator alters expression of these genes. We also demonstrate that Smad1/5 is required for RA-induced differentiation by RNA interference knockdown.

**Conclusion:**

Our results demonstrated that E8 medium is able to maintain cBC growth for weeks and RA treatment induced germ cell differentiation of cBCs through the BMP-Smad1/5 signaling pathway.

**Electronic supplementary material:**

The online version of this article (doi:10.1186/s12896-017-0332-y) contains supplementary material, which is available to authorized users.

## Background

Transgenic chickens have many applications due to their physiological characteristics. Transgenic chickens are considered a powerful bioreactor for the production of proteins of pharmaceutical and industrial interest [1–3]. Chickens are also an excellent model for human diseases [4]. Furthermore, the chicken embryo uniquely permits observation of development through the eggshell, facilitating investigation of embryo survival and development [5]. However, methods for generating transgenic chickens carrying targeted mutations are currently difficult.

Chicken primordial germ cells (cPGCs) are an efficient system for germline transmission and producing transgenic chickens [6]. Because only a limited number of cPGCs can be obtained from each embryo, the establishment of a long-term culture system for stable cPGC lines would be indispensable. However, this process is both technically demanding and resource intensive. An alternative is culturing chicken blastodermal cells (cBCs), which can be easily obtained from fertilized eggs and manipulated. cBCs are derived from the area pellucida of Eyal-Giladi and Kochav (EG&K) stage X chicken embryos [7, 8]. Somatic and germline chimeras were produced after freshly isolated cBCs were transplanted into the sub-germinal cavity of stage X (EG&K) embryos [8, 9]. Unlike mouse embryonic stem cells (mESCs), which are derived from the inner cell mass of the blastocyst, cBCs cultured in vitro have extremely low germline transmission competence. It seems that the maintenance of germline competence of cBCs is sensitive to culture conditions, as germline competence rapidly and dramatically diminishes after culture in vitro [10, 11]. Therefore, a method for inducing cBCs to enhance or to recover germline competence would be desirable for generating transgenic chickens. Indeed, overexpression of *Cvh* (chicken *Vasa* homologue) in in vitro cultured chicken ESCs derived from cBCs is able to restore germline competence by both inducing the germ cell differentiation program and impairing the somatic differentiation program [12]. Moreover, overexpression of ectopic transcription factors (*Oct4, Nanog, Sox2, Lin28, Kif4*, and *C-myc*) in chicken embryonic fibroblasts leads to the generation of induced cPGCs, which can migrate to the embryonic gonad after injection into the vasculature of Hamburger and Hamilton (H&H) stage 15 embryos [13, 14]. Compared with ectopic overexpression of transcription factors, chemical induction of pluripotency is more convenient to use for differentiation of mESCs and cESCs. However, whether chemical inducers are able to enhance cBC germline competence in vitro is unknown.

Retinoic acid (RA), an active metabolite of vitamin A, alters the expression of target genes to regulate many different growth and differentiation processes during embryogenesis and organogenesis [15]. Because RA treatment results in the rapid expression of germline-specific genes, it is the most commonly used reagent during in vitro germ cell differentiation of pluripotent cells [16]. Among the genes influenced by RA treatment are members of the Bone morphogenetic protein (BMP) family. BMP2, BMP4 and BMP8b activate Smad1/5 in the PGCs. The BMP-Smad1/5 signaling pathway is essential for the specification and proliferation of PGCs [17–19]. RA has previously been shown to promote germ cell differentiation in mESCs by activates the BMP-Smad1/5 signaling pathway [20]. However, it is unknown whether similar mechanisms of inducing differentiation exist in cBCs.

A variety of culture systems have been developed for both self-renewal or directed differentiation of pluripotent cells. One of these, E8 medium, contains only seven additional completely defined and xeno-free ingredients supplementing the standard DMEM/F-12 medium [21]. It is a feeder-free medium that supports the culture of human induced pluripotent stem (iPS) cells and mESCs [22]. Cells grown in E8 medium are appropriate to investigate molecular mechanisms because E8 medium provides a clean background detecting the accumulation of specific growth factors during the process of cell differentiation. Despite the fact that E8 medium was originally developed for mammalian systems, culturing cBCs in E8 medium is possible because the growth requirements of pluripotent mammal and chicken cells are nearly identical.

In this study, we describe the growth process of cBCs in E8 medium and demonstrate that RA treatment stimulated expression of germ-specific genes. Moreover, we reveal that RA activates the BMP-Smad1/5 signaling pathway in cBCs and this pathway is required for expression of germ-specific genes.

## Results

### Morphology and growth of cBCs in E8 medium

**Fig. 1:**
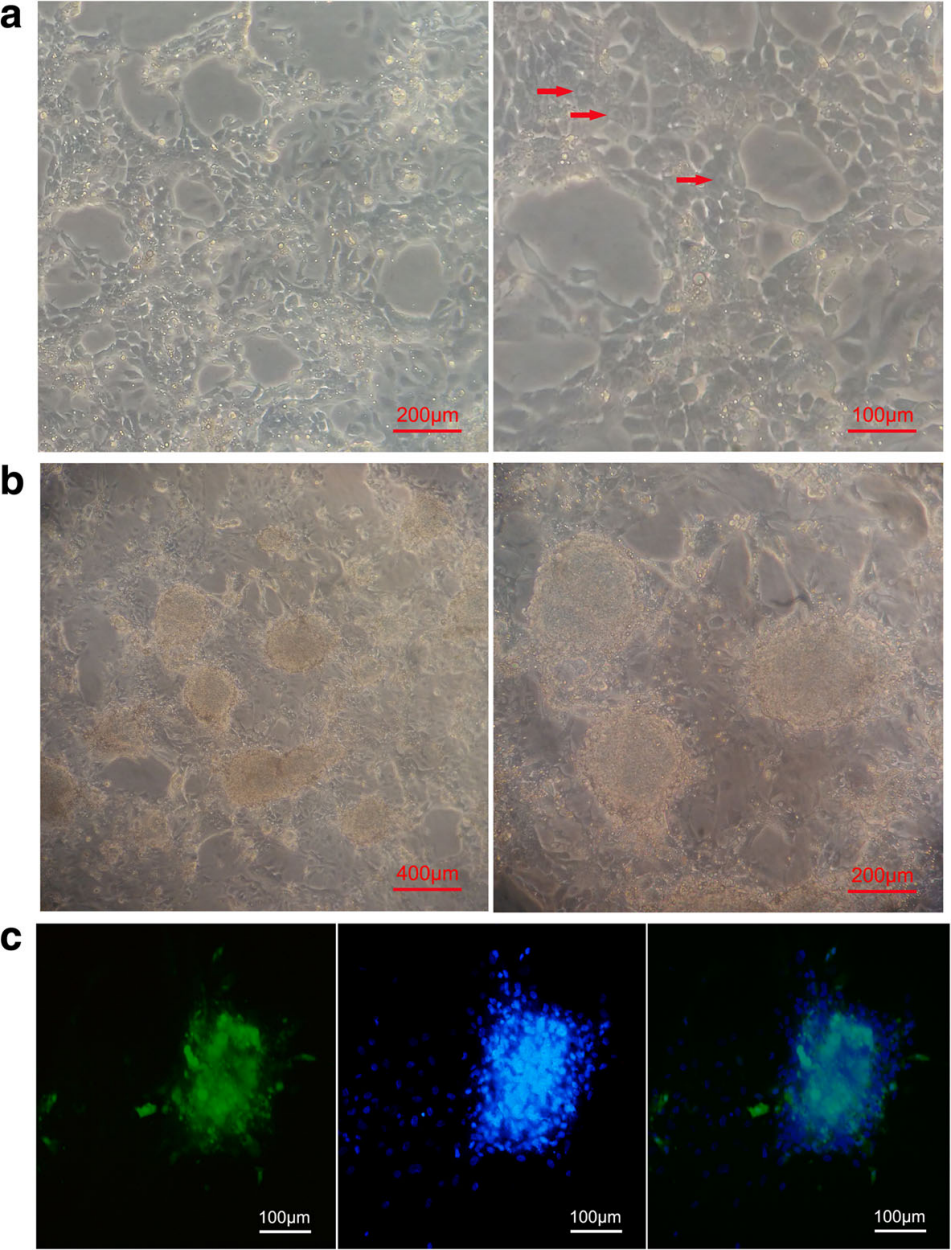
The growth of chicken blastodermal cells (cBCs) in E8 medium. **a** The cells have large nuclei and pronounced nucleoli (*arrows*), and grow in a monolayer with clearly distinguishable individual cells. **b** The cells form tight and compact colonies of multilayer cells arranged in clusters after FGF9 and TGF-β are withdrawn. **c** Immunofluorescence staining of SSEA-1 (*green*), nuclei were counterstained with DAPI (*blue*)

After cBCs were seeded, they attached firmly to culture plates within 12 h. The cells had large nuclei and pronounced nucleoli and grew in monolayer colonies with clearly distinguishable individual cells (Fig. 1a). This morphology is similar to that described previously [10]. When we withdrew FGF2 and TGF-β from E8 medium, cBCs formed tight, compact colonies of multilayered cells arranged in clusters within 48 h (Fig. 1b). We speculate that colonies with this cobblestone morphology are analogues of embryoid bodies.

In general, cBCs retained monolayer morphology for three or four passages (approximately 2 weeks). Beyond this period, most of the cells tended to grow in multilayered morphologies and the monolayer cells tended to developed fibroblast-like features. Though multilayer cells were still positive for the pluripotency marker SSEA-1 (Fig. 1c), cells developed vacuoles and stopped growing after five or six passages (approximately 3 weeks).

### LIF contributed to proliferation and pluripotent character of cBCs

**Fig. 2:**
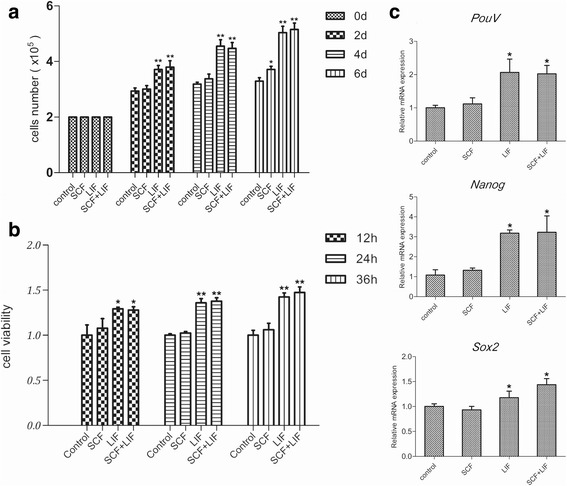
Effect of SCF and LIF on growth of cBCs in E8 medium. Cell number (**a**) and cell viability (**b**) were measured after treatment for the indicated times with SCF, LIF or both. Values are the mean ± SEM (*n* = 5). **c** The expression levels of the pluripotency genes *PouV*, *Nanog* and *Sox2* were measured by qRT-PCR after growth factor treatment for 24 h. Values are the mean ± SEM (*n* = 3). Data are representative of results in three independent experiments, and each condition is normalized to *β-actin* abundance. *, *p* < 0.05; **, *p* < 0.01

To identify the essential growth factors for culture of cBCs, we cultured cBCs under combinations of SCF and LIF, which are commonly used to enhance self-renewal and proliferation of various chicken pluripotent cells [23]. The total number of cBCs increased in the presence of LIF. However, SCF did not influence the proliferation of cBCs (Fig. 2a). The result was consistent with the cell viability essay (Fig. 2b). Following this, we examined the expression of pluripotent markers using quantitative PCR analyses. In chickens, POUV, SOX2, and NANOG are considered the core transcriptional regulators of pluripotency [24]. We detected that mRNA expression of *PouV* and *Nanog* were increased by LIF addition, and *Sox2* mRNA accumulation increased with co-addition of LIF and SCF (Fig. 2c).

These results indicate that LIF addition optimizes the effectiveness of E8 medium for culturing cBCs. Even though optimized E8 medium is unable to maintain a long-term culture for cBCs, it could be appropriate as a system for directional inducing.

### RA causes early expression of germ-specific genes in cBCs

To identify the extent of germ cell differentiation in cBCs, we measured the mRNA expression of germ-specific genes. *Stra8* (stimulated by retinoic acid gene 8) is a key intrinsic gene in meiotic initiation in response to extrinsic signaling [25]. *Dazl* (Deleted in azoospermia) is likely exclusively expressed in chicken germ cells [26, 27] and is involved in chicken gametogenesis [28]. *Cvh* is a specific marker of differentiating germ cells between the late migration stage to the post-meiotic stage [12].

**Fig. 3:**
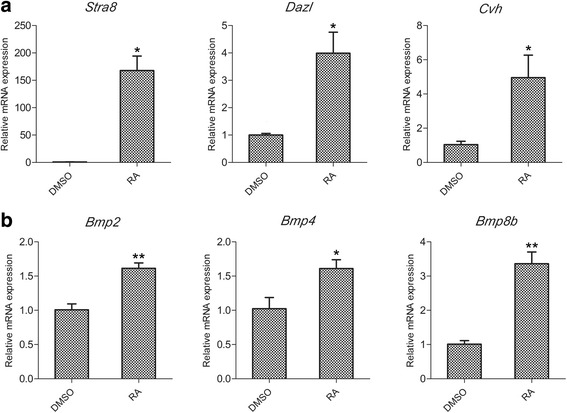
The mRNA levels of the germ cell-related genes and BMP-encoding genes during RA-induced differentiation of cBCs. The expression levels of the germ-specific genes *Stra8*, *Dazl,* and *Cvh* (**a**) and the BMP family members *Bmp2*, *Bmp4* and *Bmp8b* (**b**) were measured by qRT-PCR following 24 h of RA treatment. Data are representative of results in three independent experiments, values are the mean ± SEM (*n* = 3) and each condition is normalized to *β-actin* abundance. *, *p* < 0.05; **, *p* < 0.01

We treated cBCs with RA while they were growing in monolayer colonies. As a result of RA treatment, the mRNA expression of *Stra8* increased over 100-fold. *Dazl* and *Cvh* mRNA levels were increased approximately 4–6-fold (Fig. 3a). The BMP-Smad1/5 signaling pathway is involved in germ cell differentiation in mESCs [20], and we detected a substantial increase in *Bmp2*, *Bmp4* and *Bmp8b* mRNA expression (Fig. 3b) after RA treatment. The mRNA levels of the germ cell-related genes and BMP-encoding genes suggested that RA treatment induced cBCs to differentiate to germ cells.

### Smad1/5 activation is required for RA-mediated germ-specific gene expression

**Fig. 4:**
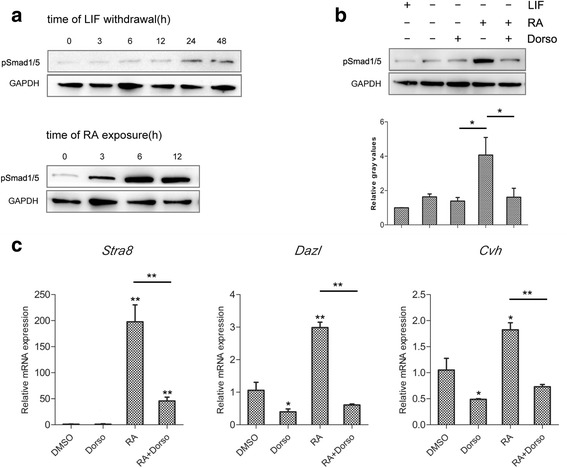
Inhibition of Smad1/5 phosphorylation decreased RA-induced germ cell differentiation of cBCs. **a** Cells were grown for the indicated time following LIF withdrawal in the presence of RA. Western blots were used to analyze the expression levels of phosphorylated Smad1/5 (pSmad1/5) and a loading control (GAPDH). Similar results were obtained in two separate experiments. **b** The expression levels of pSmad1/5 were analyzed after 12 h of RA treatment, 2 h of dorsomorphin (Dorso, inhibitor of BMP signaling pathway) treatment, or both. Similar results were obtained in two separate experiments. **c** The mRNA expression levels of *Stra8*, *Dazl* and *Cvh* were measured after RA or Dorso treatment by qRT-PCR. Data are representative of results in three independent experiments, values are the mean ± SEM (*n* = 3) and each condition was normalized to *β-actin* abundance. *, *p* < 0.05; **, *p* < 0.01

We then explored the molecular mechanisms of germ cell differentiation of cBCs. We investigated whether the activation of Smad1/5 is linked to RA treatment of cBCs. To eliminate interference of LIF, we first performed a time-course analysis of Smad1/5 activation using western blot following withdrawal of LIF from E8 medium. There is no obvious change in the levels of phosphorylated-Smad1/5 until 24 h following LIF withdrawal (Fig. 4a). We then measured the effect of RA treatment on Smad1/5 activation in E8 medium without LIF. Phosphorylation of Smad1/5 was first observed 3 h after treatment with RA and increased dramatically after 6 h. When cBCs were treated with the Smad1/5 phosphorylation inhibitor dorsomorphin [29], RA-induced phosphorylation of Smad1/5 was decreased (Fig. 4b). Additionally, inhibition of Smad1/5 phosphorylation substantially decreased the mRNA expression levels of *Stra8*, *Dazl* and *Cvh*, even in the presence of RA (Fig. 4c). These results indicate that RA promotes Smad1/5 phosphorylation, which is required for differentiation of cBCs to germ cells.

**Fig. 5:**
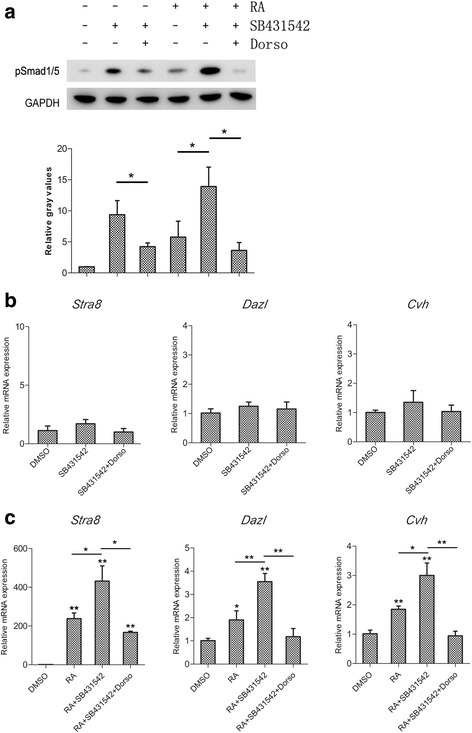
Activation of Smad1/5 phosphorylation enhanced the RA-induced germ cell differentiation of cBCs. **a** Western blots were performed after 12 h of RA treatment, followed by 2 h of Dorso or SB431542 (Activator of Smad1/5 phosphorylation) in the absence of RA. Similar results were obtained in two separate experiments. The mRNA expression levels of *Stra8*, *Dazl* and *Cvh* were measured after Dorso or SB431542 treatment in the absence (**b**) or presence (**c**) of RA. Data are representative of results in three independent experiments, values are the mean ± SEM (*n* = 3) and each condition is normalized to *β-actin* abundance. *, *p* < 0.05; **, *p* < 0.01

To determine the details of Smad1/5 activation in RA-induced germ cell differentiation, we treated cBCs with or without RA and SB431542. SB431542 is considered an inhibitor of TGF-β receptors (ALK4/5/7) in previous work [30, 31], but more recent studies have shown that SB431542 also activates Smad1/5 phosphorylation [20, 32], and this effect is not due to the direct pharmacological modulation of BMP receptor kinase activity but rather to some indirect approach [32]. We detected that SB431542 treatment increased phosphorylated Smad1/5 in response to all treatments, while the addition of dorsomorphin neutralized the effect of SB431542 (Fig. 5a). However, the activation of Smad1/5 mediated by SB431542 failed to increase germ-specific gene expression in the absence of RA (Fig. 5b). Co-treatment with SB431542 and RA increased the expression of germ-specific genes compared with RA treatment alone, and this increase was significantly reduced by dorsomorphin treatment (Fig. 5c). Moreover, the expression levels of *Smad1* and *Smad5* did not significantly change after RA, Dorso or SB431542 treatment (Additional file 1: Figure S1). According to these results, we conclude that RA activates the BMP-Smad1/5 signaling pathway, and cooperates with phosphorylated Smad1/5 to induce germ cell differentiation in cBCs.

### RNA interference of Smad1/5 reduced RA-mediated induction of germ-specific genes

**Fig. 6:**
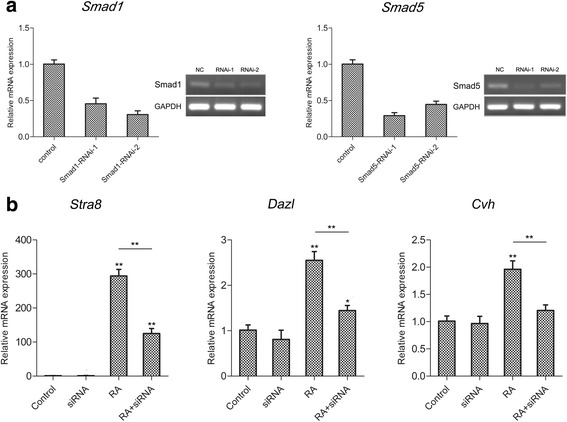
RNAi of *Smad1/5* reduces RA-mediated induction of germ cell-specific gene expression. **a** *Smad1* and *Smad5* mRNA expression levels in siRNA-infected cBCs were determined by qRT-PCR and semi-quantitative PCR. Values are the mean ± SEM (*n* = 3) and each condition is normalized to the abundance of *β-actin* as determined by qRT-PCR. **b** After co-infecting *Smad1* and *Smad5* siRNA, the mRNA expression levels of *Stra8*, *Dazl* and *Cvh* were measured in the absence or presence of RA. Data are representative of results in three independent experiments, values are the mean ± SEM (*n* = 3) and each condition is normalized to *β-actin* abundance. *, *p* < 0.05; **, *p* < 0.01

We used RNA interference to determine if *Smad1/5* is necessary for RA-mediated differentiation of cBCs. The siRNAs targeting *Smad1* and *Smad5* were efficient when we transfected them into cBCs and detected *Smad1* and *Smad5* mRNAs (Fig. 6a). We then co-transfected the most efficient siRNAs targeting *Smad1* and *Smad5*, respectively. We observed that knockdown of *Smad1/5* substantially reduced germ-specific gene mRNA expression in the presence of RA (Fig. 6b). These data are consistent with the results of dorsomorphin treatment and indicate that the BMP-Smad1/5 signaling pathway is involved in RA-induced cBC differentiation.

## Discussion

Germline competence represents the ability of transplanted cells to produce functional gametes and transmit genetic information to the next generation. Pluripotent cells that are germline competent are important tools for animal biotechnology that increases human welfare as well as elucidates basic biological phenomena. In chickens, cBCs are considered pluripotent cells because they can give rise to all somatic tissues as well as the germline after injection into the sub-germinal cavity of stage X (EG&K) recipient embryos. Chicken embryonic stem cells (cESCs) can be derived from cBCs after long-term in vitro culture beyond 20 passages [33]. In vitro, cESCs demonstrate pluripotency by differentiating into derivatives of all three germ layers via embryoid body formation [11] or monolayer culture [34, 35]. However, while cESCs efficiently contribute to somatic tissues, they fail to form germline in vivo. Therefore, cESCs are considered more similar to epiblast stem cells (EpiSCs) than to mESCs [36]. Interestingly, a recent report revealed that cESCs show greater similarity with mESCs than mEpiSCs at the transcriptome level using microarray analysis, while cBCs show a highly similar profile of gene expression as cESCs [37]. In this study, we attempted to explore whether the germline competence of cBCs can be enhanced using a protocol resembling that of germ cell differentiation in mESCs. RA is the most commonly used reagent to induce germ cell differentiation in vitro because it stimulates the expression of germ-specific genes. As expected, we detected a rapid increase in *Stra8*, *Dazl* and *Cvh* mRNA expression in cBCs after treating with RA, the results showed that cBCs were susceptible to germ cell fate induction, similar to mESCs. It is worth noting that RA is also used as reagent to induce neural phenotypes in various stem cells in vitro, and BMPs inhibition promoted neural induction [38]. We detected the increased level of BMPs in cBCs after treating with RA, and we speculated this may make cBCs tend to differentiate into germ cells rather than neurons. Nevertheless, the relationship between RA, BMPs, and neural phenotype in cBCs needs further investigation.

Developing systems perform a dynamic balance between cell proliferation and differentiation. In general, RA is considered a differentiation-inducing molecule that inhibits cell proliferation. RA-regulated processes of developmental biology include somitogenesis, neurogenesis, limb development, and visceral organ formation [38–40]. Moreover, RA is required for initiation of meiosis in vertebrate germ cells [41]. In chickens, it has been established that RA regulates the switch between mitosis and meiosis in embryonic germ cells [25]. The molecular mechanism of RA action is complex. RA activates two families of RA receptors, nuclear RA receptors (RARs) and nuclear retinoid X receptors (RXRs). RAR-RXR dimers bind to RA response elements (RAREs) and then control the transcription of nearby genes. In addition to its classical genomic effects, RA also has extranuclear and non-transcriptional effects. RA induces the rapid and transient activation of kinase signaling pathways, and then RARs and other co-regulators are phosphorylated to integrate the classical genomic effects [42].

As they lack RAREs, RA possibly regulates *Dazl* and *Cvh* through an indirect pathway in cBCs. Our results showed that the BMP-Smad1/5 signaling pathway was required for this indirect pathway. Interestingly, even though the promoter of *Stra8* contains two putative RAREs [43], *Stra8* expression decreased nearly three quarters when the BMP-Smad1/5 signaling pathway was blocked. Thus, it seems that the indirect pathway of RA activation plays an important role whether RAREs are present or not. The details of RA activation of the BMP-Smad1/5 signaling pathway still require further investigation. We speculate that Smad1/5 is activated by an increase of BMPs secretion after RA treatment. Additionally, Smad1/5 may also be activated by RA-mediated kinase signaling pathways. Indeed, RA activates p42/p44MAPKs in neurons and ESCs [44, 45], and p42/p44MAPKs enhance Smad1 phosphorylation [46].

Inducing pluripotent cells into specific cell types by the formation of embryoid bodies or monolayer culture are two commonly used methods. E8 medium supports human iPSC growth in monolayers and maintains their pluripotency beyond 50 passages. After withdrawing FGF2 and TGF-β, iPSCs form embryoid bodies [47]. For the first time, we describe the growth of cBCs in E8 medium. In E8 medium, cBCs grew in monolayer colonies and demonstrated morphology similar to cESCs. Moreover, analogues of embryoid bodies rapidly formed after we withdrew FGF2 and TGF-β from E8 medium. Recent reports revealed that the monolayer culture-mediated method of induction is simple and reliable in cESCs [34, 35], so we chose this method for cBCs induction. Even though cBCs showed the classic dynamic of pluripotent cell growth for just a limited period of time in E8 medium, this could still be appropriate as a system for directional inducing, because we cultured cBCs with a monolayer morphology for nearly 2 weeks, which is sufficient for germ cell differentiation [48–50].

A variety of media are used to culture cBCs. For example, cBCs were cultured on inactivated STO feeder cells in embryonic stem cell medium (ESA) containing growth factors including FGF2, IGF-1, SCF, IL-6, IL-11, CNTF, OSM and LIF [51]. In some reports, BRL conditioned medium was added, which contains LIF and some undefined cytokines [10, 34]. Due to the complex and undefined ingredients from feeder cells and BRL-conditioned medium, the evaluation of individual growth factors is impossible to undertake. Because E8 medium provides a clean background, we were able to evaluate the proliferation and self-renewal effect of growth factors including LIF and SCF. LIF influenced the proliferation and pluripotency of cBCs. This was in accordance with the observation that the LIF-STAT3 signaling pathway is a hallmark of pluripotency in both human and mouse ESCs [52]. Surprisingly, SCF addition did not have an effect on proliferation or pluripotency. We speculate that SCF may play a role in pluripotent cells by cooperating with other growth factors. Therefore, additional growth factors should be systematically tested to evaluate their efficiency and map the network of cross talk between them.

Although injection lentiviral vectors into subgerminal cavity to produce chicken chimeras is highly efficient, lentiviral transduction does not allow targeted gene editing because the transgene inserts randomly at multiple sites [53]. In contrast, using homologous recombination or transcription activator-like effector nucleases (TALEN) to transduce targeted mutations in cPGCs, targeted gene knockout chickens have been produced [54, 55]. However, in the decades that have passed since the first PGC-mediated chicken chimeras were produced, only a few laboratories have been able to culture cPGCs. Though it is not very efficient, cBC-mediated transgenic chicken production is valuable because cBCs are much more easily obtained and manipulated than cPGCs [56]. By inducing cBCs to differentiate into germ cells in vitro, we may enhance the efficiency of cBC-mediated transgenic chicken production.

## Conclusions

In this study, we demonstrated that E8 medium is able to maintain cBC growth for weeks. We also revealed that RA treatment induced germ cell differentiation of cBCs through the BMP-Smad1/5 signaling pathway. These results will assist our understanding of the complicated molecular mechanism of RA action. Because the germline competence of cBCs may be enhanced through RA treatment, induced cBCs could be important alternatives in the production of transgenic chickens.

## Methods

### Isolation and culture of cBCs

The area pellucida was isolated from Stage X (EG&K) embryos of Hyline chicken (*Gallus gallus*) as previously described [33], washed twice with DMEM/F-12 medium (Gibco, Carlsbad, CA) and dispersed gently using a 1000-μl pipette into a single cell suspension. The cells were centrifuged at 350× g for 5 min, the supernatant was removed, and cells were resuspended in complete E8 medium (Gibco) containing 100 U/ml penicillin and 100 μg/ml streptomycin (Gibco). The cells were seeded at a density of 2 × 10^4^ cells/cm^2^ in Vitronectin (Gibco)-coated culture plates (Costar, Corning, NY). When cells grew to 70–80% confluence, passaging was performed by washing and pipetting the cells with Ca^2+^/Mg^2+^-free phosphate buffered saline (PBS; Gibco).

### Immunofluorescence detection

Adherent cells were washed with PBS and fixed in 4% paraformaldehyde at room temperature for 20 min, permeabilized with 0.5% Triton X-100 (Sigma-Aldrich, St. Louis, MO) in PBS for 10 min. After incubation with blocking buffer containing 5% newborn bovine serum (BSA, Solarbio, Beijing, China) for 20 min, the cells were then stained with anti-SSEA-1 antibodies (1:100, Abcam, Cambridge, UK) at 4 °C overnight and at rewarmed at 37 °C for 45 min. Alexa Fluor 488 goat anti-mouse IgG (1:1000, Abcam) was added and incubated at room temperature for 1 h. The nuclei were stained with 10 μM DAPI (Sigma-Aldrich) for 30 min. The fluorescence images were obtained with fluorescence microscopy (IX71, OLYMPUS, Tokyo, Japan).

### Treatment of cultured cells with the drugs

The cells were treated with all-trans RA (Merck Millipore, Bedford, MA) at 1 μM, dorsomorphin (Selleck, Houston, TX) at 5 μM, and SB431542 (Selleck) at 5 μM. When co-treating with RA and these inhibitors, the cells were treated with RA for 12 h, changed medium and added inhibitors to treat for another 2 h. The chemicals were dissolved in dimethyl sulfoxide (DMSO, Sigma-Aldrich) and diluted with E8 medium. The final concentration of DMSO in the medium was less than 0.1%.

### Western blot analysis

Western blotting was performed according to a previously described method [57]. Adherent cells were lysed in RIPA buffer (50 mM Tris, pH 8.0, 150 mM NaCl, 1% NP-40, 0.5% deoxycholic acid and 0.1% SDS) supplemented with Halt Protease Inhibitor Cocktail (Thermo Fisher Scientific, Waltham, MA). Lysates were centrifuged and the protein concentration of supernatants was estimated using a BCA protein assay kit (Beyotime Biotechnology, China) following the manufacturer’s instructions. After blocking with 5% BSA at room temperature for 2 h, primary antibodies directed against phosphorylated Smad1/5 (1:1000, Cell Signaling Technology, Boston, MA) and glyceraldehyde-3-phosphate dehydrogenase (GAPDH, 1:5000, Abcam) were added and the blots incubated overnight at 4 °C. Primary antibodies were detected using species-specific HRP (horseradish peroxidase)-conjugated secondary antibodies (1:5000, Cell Signaling Technology) at room temperature for 2 h.

### Reverse transcription and quantitative PCR analyses

Total RNA was isolated using TRIzol reagent (Invitrogen, Carlsbad, CA) following the manufacturer’s instructions. PrimeScript RT Master Mix reverse transcription kit (TaKaRa, Japan) was used for cDNA synthesis. Reverse transcription products were amplified with the SYBR Premix Ex Taq PCR kit (TaKaRa). PCR amplification was conducted on an automated StepOne system (Applied Biosystems, Carlsbad, CA). Relative gene expression data were analyzed with the 2^-ΔΔ^Ct method. Primers were designed by using Primer 6.0 software. Primer sequences and PCR product lengths are listed in Table 1.

**Table 1:** Primers for PCR analysis

Genes	Accession no.	Primer sequences (5′ to 3′)	Product length (bp)
*PouV*	NM_001110178	GCCAAGGACCTCAAGCACAA ATGTCACTGGGATGGGCAGA	511
*Nanog*	NM_001146142.1	CAGCAGACCTCTCCTTGACC AAGCCCTCATCCTCCACAGC	586
*Sox2*	NM_205188.2	AGGCACAGGCAACTCCAACTC GCCGAGCTGCTCTTGCATCAT	472
*Stra8*	XM_416179	GTGAGGGACAGTGGAGGTAA CAGAAATGCCGCTTGTAAAT	166
*Dazl*	NM_204218	CTGGGGAGCAAAGAAACTACG CAAAGGTGTTCCTCAGACGGT	213
*Cvh*	NM_204708	GGGAAGATCAGTTTGGTGGA GACAAAGAAAGGCTGCAAGG	388
*Bmp2*	NM_204358.1	TGGTGGAGGTGGTTCACTTGGA TGTTTGTGTTTCGCTTGACGCTTT	184
*Bmp4*	NM_205237.3	ACGAAGTGATGAAGCCGCTGTC TGATGAGTCTGTGCCTGGTGGA	196
*Bmp8b*	XM_003642583.2	AACGCCACCAACCACGCCAT CAGCCACAGGACTTCACCACCATA	176
*Smad1*	NM_001201455.1	CAACCCAACAGTCACCCGTTCC AGGCAGGTAAGCAGGAGGAGGA	156
*Smad5*	NM_001014968.1	CCAGATTCCTTCCAGCAACC GCTTGTGTCCATAGACTGAGAG	213
*β-actin*	NM_205518	GAACCCCAAAGCCAACAGA GGAGGGCGTAGCCTTCATAGA	185

### Cell viability assay

Cells were seeded into Vitronectin-coated 96-well culture plates (Costar) at a density of 5000 cells/well and grown in complete E8 medium. After 1 day of culturing, medium was carefully replaced with fresh E8 medium and treated with appropriate growth factors for 12, 24 or 36 h, respectively. Medium was then replaced with fresh E8 medium containing diluted CCK-8 reagent (1:10, Dojindo Laboratories, Japan), and incubated for 2 h at 37 °C. The CCK-8 reaction product was quantified by measuring absorbance at 450 nm using an ELx800 absorbance microplate reader (BioTek, Vermont, USA).

### RNA interference assay

The sequences of siRNAs targeting *Smad1* and *Smad5* were used previously [58] and are listed in Table 2. RNA oligonucleotides for RNAi experiments were obtained from GenePharma Inc. (Shanghai, China). When cBCs grew to 70–80% confluence in E8 medium, the cells were transfected with siRNA using Lipofectamine3000 (Invitrogen). A non-targeting siRNA was used as a negative control. After 24 h, transfection mixtures were replaced with E8 medium. After another 24 h, cBCs were collected for analysis or treated with experimental drugs as described above.

**Table 2:** The siRNA sequences

siRNA codes	Target sequences (5′ to 3′)
siRNA*-Smad1-1*	GGGCTGCTCTCCAATGTTA
siRNA*-Smad1-2*	GGATAGAGATACACCTTCA
siRNA*-Smad5-1*	TGCGACATTTCCAGATTCC
siRNA*-Smad5-2*	GGTGTTCGATTGTGTATTA
Negative control	TTCTCCGAACGTGTCACGT

### Statistical analysis

SPSS v16.0 software (SPSS Inc., Chicago, IL) was used to analyze data sets using the Student’s *t-*test. *P*-values less than 0.05 were considered significantly different and *P*-values less than 0.01 were considered extremely significantly different. All experimental data are reported as the mean and error bars represent the experimental standard error.

## Additional file

Additional file 1: Figure S1. The mRNA expression levels of *Smad1* and *Smad5* were measured after RA, Dorso and SB431542 treatment. Data are representative of results in three independent experiments, values are the mean ± SEM (*n* = 3) and each condition is normalized to *β-actin.* *, *p* < 0.05; **, *p* < 0.01. (TIF 8187 kb)
