# Supplementary figures and images for: Retinoic acid promotes expression of germline-specific genes in chicken blastoderm cells by stimulating Smad1/5 phosphorylation in a feeder-free culture system
Source: BMC Biotechnol. 2017 Feb 20;17:17. doi: 10.1186/s12896-017-0332-y (PMC5319176; doi:10.1186/s12896-017-0332-y)

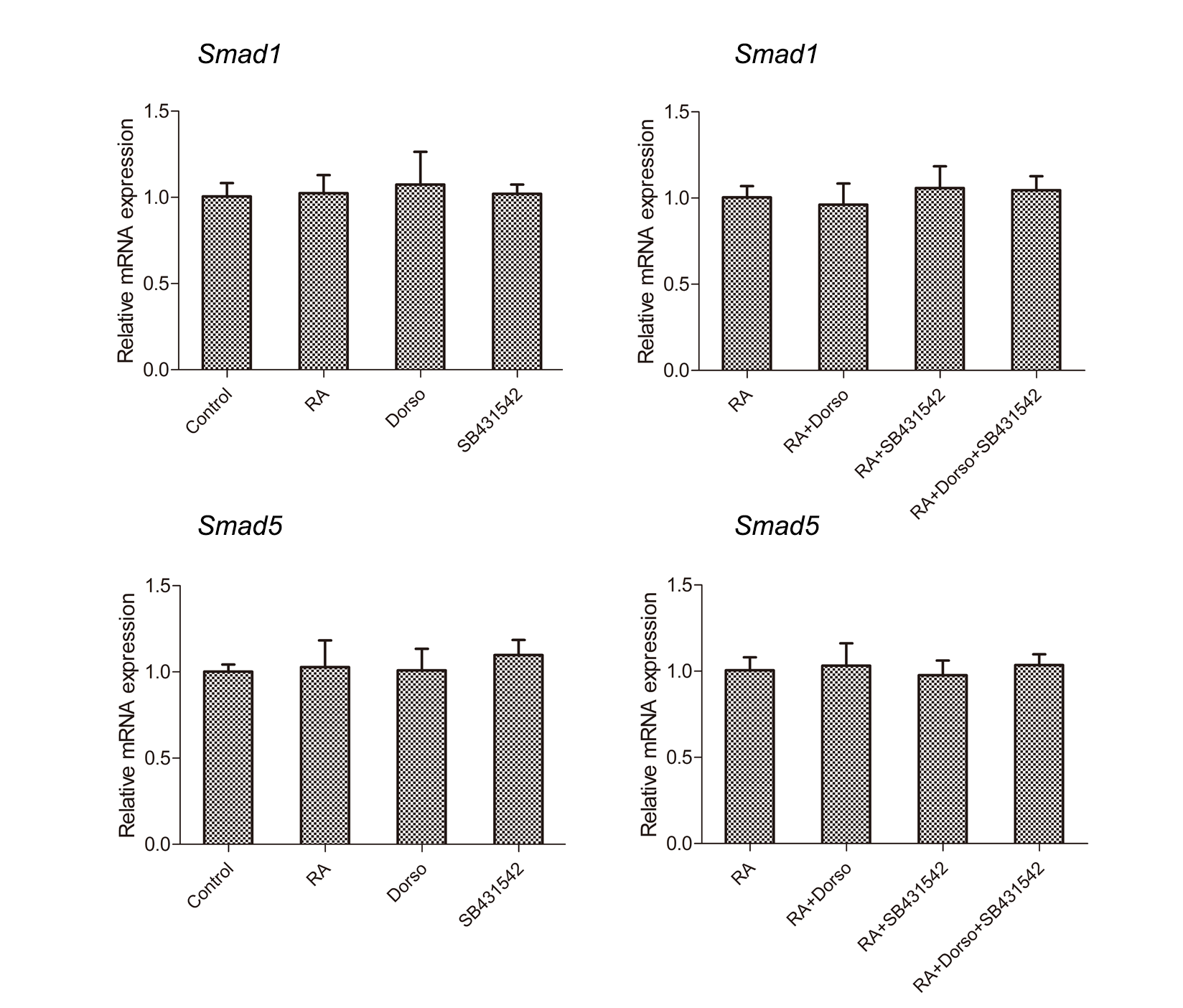

Supplement: Additional file 1: Figure S1. — The mRNA expression levels of Smad1 and Smad5 were measured after RA, Dorso and SB431542 treatment. Data are representative of results in three independent experiments, values are the mean ± SEM (n = 3) and each condition is normalized to β-actin. *, p < 0.05; **, p < 0.01. (TIF 8187 kb) [file 12896_2017_332_MOESM1_ESM.tif]
